# Application of the Semantic Fluency Test in the Screening of Mandarin-Chinese-Speaking Older Adults with Mild Dementia of the Alzheimer Type

**DOI:** 10.3390/bs13080635

**Published:** 2023-07-30

**Authors:** Ming-Ching Lin, Yu-Chun Chih

**Affiliations:** Department of Speech Language Pathology and Audiology, Chung Shan Medical University, Taichung 402, Taiwan; daminaki@gmail.com

**Keywords:** semantic fluency test, dementia, Alzheimer disease, Mandarin Chinese speakers, older adults, ROC curve

## Abstract

Semantic fluency tests have been widely used as a screening test for dementia of Alzheimer type. However, few studies have explored the application of semantic fluency tests in Mandarin Chinese speakers. This study aimed to explore the feasibility of using different semantic fluency test categories to distinguish between older adults without cognitive impairments and those with dementia of Alzheimer type in Taiwan. A total of 58 healthy older adults and 54 individuals with dementia of Alzheimer type were recruited. Semantic categories of “animals”, “fruits”, “vegetables”, “birds”, “means of transportations” and “musical instruments” were administered to participants. The scores from two groups of participants for each category were analyzed. Significant differences in the test scores of each category between two groups of participants were found. The results also revealed that the variables related to whether the participant had dementia, gender, age, and years of schooling significantly influenced the semantic fluency scores for each category. Among all the demographic characteristic of participants, the diagnosis of dementia was the most determining factor. Furthermore, this study proposed optimal cutoff points and calculated the AUC for various test durations (i.e., 30 s, 45 s and 60 s) and semantic categories in the semantic fluency test, which may serve as a reference that would help clinical personnel distinguish between older adults without cognitive impairments and those with dementia of Alzheimer type in Taiwan.

## 1. Introduction

With an increasing trend of global aging, the number of dementia cases is expected to increase annually, with dementia of the Alzheimer type (DAT) being the most prevalent type. Patients with DAT generally experience impaired language functions, the most apparent being semantic impairment, during the early stages of DAT [1,2]. Studies have reported that semantic impairment may occur before a formal diagnosis of DAT [3] and is primarily associated with deterioration in the medial temporal lobe (MTL), inferior temporal lobe (ITL), and the temporooccipital junction [4,5]. The MTL plays the primary role in long-term memory [6], including episodic memory and semantic memory. Although studies have primarily investigated the role of the MTL in the formation of episodic memories, some have proposed that the same structures are used in the formation of both episodic and semantic memory [7,8,9]. Hirni et al. [10] reported that volume reduction in the left perirhinal cortex is associated with reduced semantic performance, thus highlighting the predictive effects of physiological changes on the semantic memory of patients with DAT. Numerous clinical approaches have been developed to assess the semantic functions in patients. Of these, the semantic fluency test is a simple, convenient, and common testing tool for assessing patients with DAT. The test involves asking participants to generate as many words as possible from a given semantic category within a specified timeframe, for example, asking patients to name as many animals as they can in 60 s (s). The examiner records the participants’ responses, and the number of repetitions and unrelated words are calculated as well as the total score of correct productions.

Studies have indicated that individuals in the early stages of DAT exhibit reduced performance in the semantic fluency test [1]. Notably, Auriacombe et al. [11] observed a decline in semantic fluency in patients with DAT three to five years before diagnosis, which further considerably declines two years prior to diagnosis. This decline has been shown to continue throughout the course of the disease [2,12,13,14]. In a study on brain image examination, Ahn et al. [15] revealed a significant positive correlation between the total score of the semantic verbal fluency test and the cerebral glucose metabolism in the prefrontal, parietal, cingulate, temporal cortex, and subcortical regions. Studies have also reported that a reduction in gray matter density in the left thalamus, insula, hippocampus, and parahippocampal gyrus is associated with decreased performance in the semantic fluency test [16,17]. Scheff et al. [5] indicated that lesions may occur in the ITL during the early stages of DAT progression and revealed a significant association between the total synaptic number and volume in the ITL and the performance in the semantic fluency test, as observed in an animal-naming task (hereafter referred to as “animal fluency test”). Additionally, significant differences in the total synaptic number were reported between older adults with amnestic mild cognitive impairment and those without cognitive impairment. In summary, lesions typically occur in the hippocampus and parahippocampal gyrus of patients with DAT during the early stages of the disease, leading to impaired performance in the semantic fluency test. These regions have been closely linked to semantic fluency [8,13,18]. Consequentially, semantic fluency tests are considered a valuable tool for assessing the semantic fluency function between individuals without cognitive impairments and those with DAT.

As for the reliability and validity of the semantic fluency test, several studies have been conducted on healthy adults without cognitive impairments. Harrison et al. [19] examined 365 participants with a mean age of approximately 40 years old and administered the animal fluency test. The results of Pearson’s r analysis revealed that the semantic fluency test had a moderate test–retest correlation (*r* = 0.68) when the test was repeated within a timeframe of one to eight weeks. Bird et al. [20] tested 188 healthy participants between 39 and 75 years old and reported a moderate test–retest correlation (*r* = 0.56) over a timeframe of approximately one month. Vora et al. [21] recruited 15 healthy adults with a mean age of approximately 23 years old to evaluate the test–retest reliability of semantic fluency tests based on the naming of animals and boys’ names. The tests were administered in two sessions separated by a 10–12-day interval, with the initial test being conducted using a paper–pencil test and the second test being an oral test. Despite the difference in administration mode, the test scores demonstrated moderate correlation (*r* = 0.65) between the two sessions. For measuring the test–retest reliability of the semantic fluency test, studies have examined the presence of practice effects in participants by comparing their performance in the initial test and the retest. Bird et al. [20] reported significant practice effects in the retest of the semantic fluency test. However, these improvements were relatively small, with participants exhibiting an increase of only one or two words. Harrison et al. [19] reported that 60% of participants scored higher on the second test. However, for 25–32% of the participants, the retest scores were lower than their initial test scores, indicating that the practice effects did not occur consistently.

The test–retest reliability of the semantic fluency test in patients with DAT has been investigated in several studies. St-Hilaire et al. [22] administered two animal fluency tests to healthy older adults and patients with DAT, with a one-year interval between the tests. The results revealed a higher test–retest correlation for healthy older adults (*r* = 0.711) than for patients with DAT (*r* = 0.493). Similar findings were reported by Clark et al. [23], who assessed the performance of healthy older adults and patients with DAT in the animal fluency test. The findings revealed that the decline in semantic fluency was more pronounced in patients with DAT than in healthy older adults. However, given the one-year test–retest interval in the study, the findings may also reflect the deterioration of semantic memory in patients with DAT, leading to inconsistent retest scores. Cooper et al. [24] recruited 23 older adults without cognitive impairments, 23 patients with mild DAT, and 23 patients with mild cognitive impairments. The participants completed two animal fluency tests within a short test–retest interval of one week. The results revealed a high and moderate degree of test–retest reliability in the older adults without cognitive impairments (*r* = 0.85) and patients with DAT (*r* = 0.50), respectively. The study reported that 40% of patients with DAT demonstrated lower performance in the retest and that the test–retest correlation for patients with DAT was lower than that for older adults without cognitive impairments. Additionally, significant improvements were observed in the retest performance of patients without cognitive impairments, indicating the occurrence of practice effects. However, because no significant differences were observed between the test and retest scores of patients with DAT, the practice effects were less apparent for older adults with DAT.

The effectiveness of using the semantic fluency test for screening patients with DAT can be measured using sensitivity and specificity. St-Hilaire et al. [22] administered the animal fluency test to 62 healthy adults and 62 patients with DAT, with both groups comprising participants aged at least 50 years. The total scores were converted into Z-scores to analyze sensitivity and specificity at different cutoff values. A cutoff value of Z = −1.00 yielded the highest accuracy (80.6%), accounting for 71% and 90.3% of sensitivity and specificity, respectively. Moreno-Martínez et al. [25] collected scores from 100 patients with DAT and 49 older adult participants aged at least 50 years in 14 category fluency tasks and revealed differences in the sensitivity and specificity of the tests at optimal cutoff values. Notably, the sensitivity and specificity of the tests ranged from 82% to 99% and from 49% to 90%, respectively. The analysis also revealed that the area under the curve (AUC) ranged from approximately 85.7% to 95.2%, indicating that the category fluency tests had greater predictive power than random guessing. The semantic fluency test for the “body parts” category had the highest AUC and a sensitivity of 98% but a specificity of only 69%. Therefore, whether “body parts” is the optimal category for semantic fluency tests requires further investigation. Mok et al. [26] administered Cantonese categorical semantic fluency tests with categories of “animal”, “fruit”, and “vegetable” to 72 patients with DAT and 81 age-matched, healthy older adults in Hong Kong. The total scores in the three categories were analyzed, and cutoff values were established based on the participants’ education levels. The sensitivity, specificity, and positive predictive value of the tests ranged from approximately 86.8% to 87.5%, 73.8% to 93.4%, and 0.77 to 0.93, respectively, indicating a high positive predictive value. The authors reported that the test was convenient to administer and had satisfactory sensitivity and specificity. However, several studies have indicated potential correlations between semantic fluency scores and demographic variables. The semantic fluency scores of participants without cognitive impairments are typically negatively correlated with age but positively correlated with education level [27]. Notably, gender differences have also been found to influence test scores in various categories [25,28,29]. Kawano et al. [30] indicated that the animal fluency scores of older adults with DAT were negatively correlated with age but positively correlated with education level. This result may be explained by the cognitive reserve theory [31], which proposes that improvements in an individual’s cognitive processing ability is based on educational or occupational accomplishments, personal lifestyles, cognitive stimulation behavior, or personality, and it enables the brain to retain sufficient cognitive abilities to respond to or compensate for pathological changes when the brain is damaged. Therefore, when selecting semantic categories for semantic fluency testing, researchers should consider the potential correlations with demographic variables of the participants and make necessary adjustments to increase the validity of the findings.

The Taiwanese literature on the reliability and validity of the semantic fluency test is limited. Chung et al. [32] compared the performance of healthy illiterate older adults and patients with early DAT in the one-minute (min) semantic fluency test. However, the study did not provide details on the sensitivity, specificity, and cutoff point of the test. Moreover, the study had a small sample size and a limited research region. Further research is needed to explore this topic in more detail. Chen [33] conducted six different semantic fluency tests in one min on 220 healthy middle-aged and older adults living in northern Taiwan. The study used regression coefficients derived from demographic variables to adjust raw scores and establish percentile rank norms for the adjusted scores. The results revealed moderate-to-high test–retest correlations (0.65 < *r* < 0.79). However, because the study did not recruit patients with DAT, the findings cannot be applied to determine the validity and reliability of semantic fluency tests on older adults without cognitive impairments and patients with DAT in Taiwan.

The test duration is a crucial variable to consider when conducting the semantic fluency test. The typical test duration required to conduct the general semantic fluency test is one min. Few studies have explored the effect of different test durations on the test results. Mack et al. [34] employed a shorter test duration of 30 s to reduce participant stress. In practice, a test duration of 30 s is expected to be more feasible than that of one min. Teng et al. [35] also reported the use of a 30 s test duration for the “four-legged animals” semantic fluency test in the Cognitive Abilities Screening Instrument (CASI). A normative study on the application of the CASI in Taiwan revealed that 30 s semantic fluency tests for the category of “four-legged animals” successfully distinguished healthy older adults and patients with Alzheimer’s disease [36]. Mirandez et al. [37] compared the performance of participants with mild cognitive impairments and without cognitive impairments in semantic fluency tests in the categories of “animals”, “fruits”, and “means of transportation.” They found significant differences between the two groups for the “animal” category within the first 15 s of testing and for the “fruits” and “means of transportation” categories within the first 30 s of testing. Herrera-García et al. [38] advocated that a test duration of 30 s for the semantic fluency test can effectively distinguish participants with cognitive impairments. Accordingly, whether a test duration of one min for the semantic fluency test is necessary remains a topic for further discussion.

The purposes of the present study are as follows: (1) to investigate the differences in semantic fluency test performance between healthy older adults and patients with DAT in Taiwan; (2) to examine the effects of demographic characteristics on semantic fluency scores; and (3) to identify differences in semantic fluency scores obtained at different test durations. Additionally, this study established cutoff points for each category of semantic fluency tests with different test durations and test–retest reliability for semantic fluency tests.

## 2. Materials and Methods

### 2.1. Research Participants

This study recruited a total of 112 participants and divided them into two groups: a healthy group of 58 healthy older adults (28 men; 30 women) and a DAT group of 54 patients with mild DAT (23 men; 31 women). The following participants were included in the healthy group: (a) those aged at least 65 years; (b) those who spoke Mandarin Chinese as their main language; and (c) those who lived in Taiwan for the past five years. The exclusion criteria for the healthy group were as follows: (a) a diagnosis or history of stroke, brain damage, dementia, or other diseases related to the nervous or mental system; (b) inability to follow commands due to visual or hearing impairments; (c) inability to pass the Mini-Mental State Examination [39] (MMSE; those with seven or more years of schooling and a score lower than 24; those with six or less years of schooling and a score lower than 21; and those with no schooling experience or who are illiterate and have a score lower than 16 [40]); and (d) at least a total score of two in the Ascertain Dementia 8 [41] (AD8) questionnaire for cognitive screening [42]. The following participants were included in the DAT group: (a) those aged at least 65 years; (b) those who spoke Mandarin Chinese as their main language; (c) those who lived in Taiwan for the past five years; and (d) those who had records from their residing hospital or their family members detailing a probable diagnosis of AD by neurologists or psychiatrists, particularly those with a diagnosis of mild DAT or those with a total score of one on the Clinical Dementia Rating Scale (those with a score of 0.5 were excluded). The exclusion criteria for the DAT group were as follows: (a) a diagnosis or history of stroke, brain damage, or other non-DAT diseases related to the nervous or mental system; and (b) inability to follow commands due to visual or hearing impairments. The demographic characteristics data of the two groups are presented in Table 1. There was a significant difference in age between two groups (*p* < 0.001), and no significant differences were observed in the years of schooling between two groups.

### 2.2. Research Materials

1. MMSE
  The MMSE is a common clinical screening tool for Alzheimer’s disease and can also be applied for the rapid screening of cognitive impairments. The MMSE cutoff points for different years of schooling were adopted from Chang and Tsai [40]. For participants with no schooling experience, a score of 15 or below indicated cognitive impairment. Participants with one to six years of schooling experience (i.e., elementary school education level) were considered cognitively impaired if they scored 20 or below. Finally, participants with at least seven years of schooling (i.e., middle school and above education level) were classified as cognitively impaired if they scored 23 or below on the MMSE.
2. AD8
  The AD8 questionnaire, developed by Washington University in St. Louis, is a widely used brief cognitive screening tool. The questionnaire can be self-administered by participants and has a cutoff point of 1/2 for distinguishing participants without cognitive impairments and those with mild cognitive impairments. Yang et al. [42] administered the AD8 questionnaire to Taiwanese participants by using a cutoff point of 1/2 and reported a sensitivity and specificity of 95.9% and 78.1%, respectively. The AD8 questionnaire offers a convenient and efficient means of distinguishing older adults in the mild cognitive impairment stage of dementia from those without cognitive impairments.
3. Semantic fluency tests
  This study referenced semantic categories used in the literature, namely “animals”, “fruits”, “vegetables”, “birds”, “means of transportations”, and “musical instruments” for the semantic fluency test. The tests were arranged in a pseudo-random sequence. To prevent participants from being influenced by words listed in the “birds” category when listing words in the “animals” category, testing with the “animals” category was performed before testing with the “birds” category.

### 2.3. Procedures

The test locations of this study included community activity centers, daycare centers, hospitals, long-term care institutions, nursing institutions, and participants’ residences. The tests were conducted in quiet and well-illuminated spaces to minimize any potential interference or distractions. The tests were individually administered, with the participant and the researcher seated across or beside each other. Participants were positioned to face the direction with less interference to enhance focus.

Prior to the commencement of the study, all participants or their legal representatives were required to sign a consent form approved by the Human Research Institutional Review Board of a medical center. After obtaining consent, the participants’ demographic data, including age, gender, and years of schooling, were collected. For older adults with DAT, detailed information regarding the diagnosis content and severity was recorded. Older adults without cognitive impairments were administered the MMSE and AD8 questionnaire. After the diagnosis records were acquired and testing with MMSE and AD8 was completed, semantic fluency tests were administered to participants who fulfilled the inclusion criteria, and the test process was audio-recorded.

During the tests, participants were guided with specific instructions: “Please list as many (semantic category) as you can think of before time is up. Responses cannot be repeated. Please respond in Mandarin Chinese. You may begin.” Each participant was given one min to generate responses for each semantic category, and the participants’ responses were recorded and scored at the 30, 45, and 60 s time points. After testing, the total number of correct responses that fit each category was calculated for each participant, and each correct response was assigned one point. The participants’ total scores for the 30, 45, and 60 s time points were recorded. However, under the following circumstances, no point was given to the participants’ response: (1) the response was repeated; (2) the response was unrelated to the category (e.g., responses of vegetable names during the test with the “animals” category); (3) the response was the name of a mythological or fictional character (e.g., a response of “Donald Duck” during the test with the “animals” category); (4) the only difference between the responses was the adjective (e.g., responses of “black dog” and “white dog” only earns the participant one point); and (5) unclear responses or responses that did not exist. Additionally, to establish test–retest reliability, 29 participants were selected from the healthy group and 30 participants were selected from the DAT group for retests that were conducted two to four weeks after the initial test.

### 2.4. Data Analysis and Statistical Methods

An independent sample *t*-test was performed to compare the differences in the scores for each category between the two groups. The results of the independent sample *t* test were then subjected to statistical analysis to compare the participants’ scores at different test durations (e.g., 30, 45, and 60 s) for each category and to determine whether significant differences existed between the performance of the healthy group and the DAT group (*p* < 0.05).

Subsequently, multiple regression analysis was conducted to determine the correlations between the participants’ demographic variables (e.g., whether the participant had DAT, gender, age, and years of schooling) and test scores (*p* < 0.05).

Validity analysis was conducted to evaluate the discriminatory power of each category of the semantic fluency test in distinguishing between the healthy group and the DAT group. The Youden index (*J*) [43] was used to obtain the optimal cutoff point for each category. Subsequently, the sensitivity, specificity, and AUC of each category at the optimal cutoff point at different test durations were obtained.

In the reliability analysis, Pearson product–moment correlation coefficient analysis was used to examine the test–retest reliability. The analysis results were interpreted using the explanations provided in Akoglu [44]: 0.1 < *r* < 0.39, 0.4 < *r* < 0.69, and 0.7 < *r* < 0.99 indicate weak, moderate, and strong correlation, respectively.

## 3. Results

### 3.1. Test Scores of the Two Group of Participants

The independent sample *t*-test analysis revealed significant differences (*p* < 0.001) in the semantic fluency scores between the healthy group and the DAT group for each category at different test durations, as presented in Table 2.

### 3.2. Relationships between Demographic Variables and Semantic Fluency Scores for Different Categories

Table 3, Table 4 and Table 5 present the results of the multiple regression analysis of the effect of demographic variables on the semantic fluency scores for each category at test durations of 60, 45, and 30 s, respectively. The results revealed that the variables related to whether the participant had DAT, gender, age, and years of schooling significantly influenced the semantic fluency scores for each category. Whether the participant had DAT exhibited a significant relationship with semantic fluency scores for each category regardless of the test duration (*p* < 0.001). Participants with DAT achieved lower test scores. Additionally, the β value of whether the participant had DAT was larger than that of other demographic variables, indicating a stronger correlation with test scores. The relationships of the other demographic variables with semantic fluency scores for each category varied, indicating that among the four demographic variables, whether the participant had DAT exerted the greatest and most consistent influence on semantic fluency scores.

### 3.3. Establishment of Test Validity

**Figure 1:**
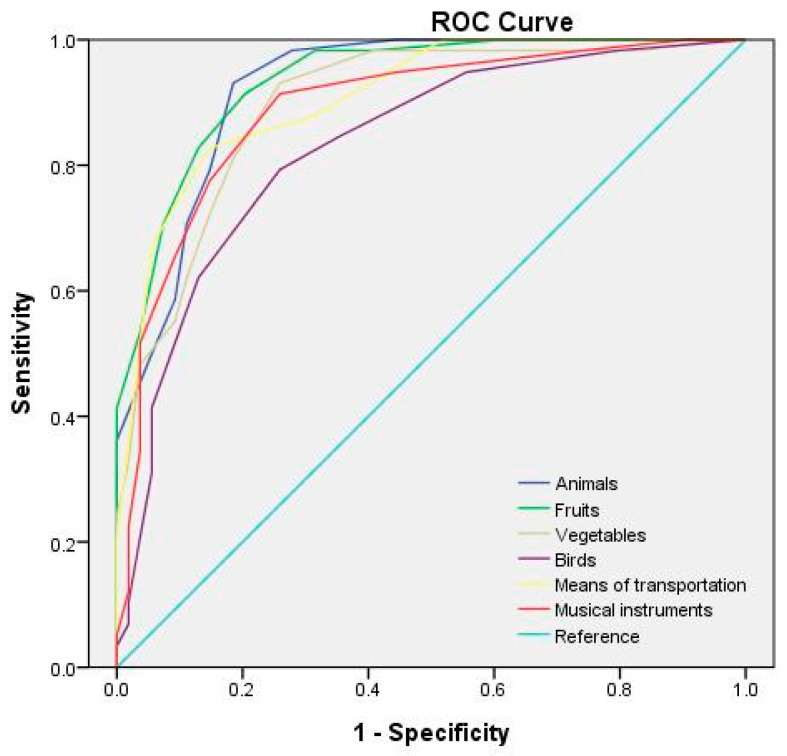
ROC curves for the semantic categories for a 60 s test duration.

**Figure 2:**
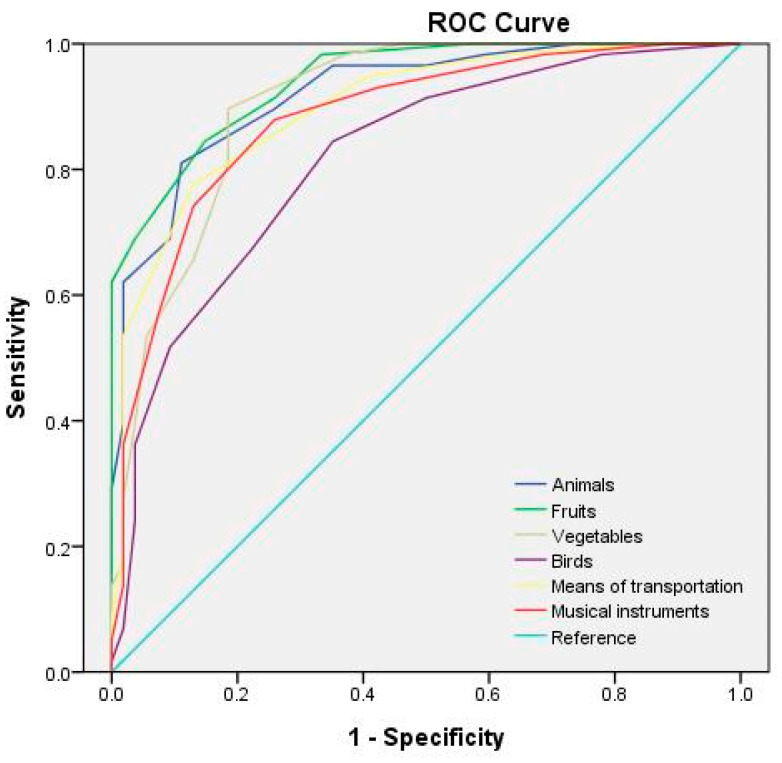
ROC curves for the semantic categories for a 45 s test duration.

**Figure 3:**
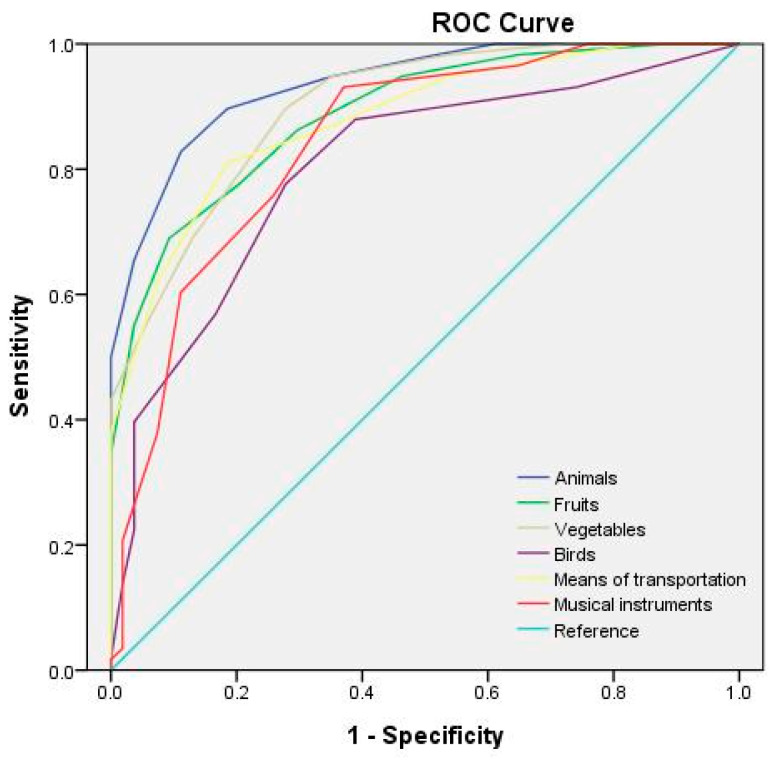
ROC curves for the semantic categories for a 30 s test duration.

Table 6 lists the optimal cutoff points and AUCs for each category of the semantic fluency test at different test durations. The receiver operating characteristic (ROC) curves for the semantic fluency test at test durations of 60, 45, and 30 s are depicted in Figure 1, Figure 2 and Figure 3, respectively. The study adopted the interpretation and ratings of AUC proposed in Mandrekar [45]. An AUC of >0.90 is considered outstanding (e.g., the 60 s fruit, animal, and means of transportation fluency test; the 45 s fruit, animal, and vegetable fluency test; and the 30 s animal fluency test), a score between 0.8 and 0.9 is considered excellent (e.g., the 60 s vegetable, musical instrument, and bird fluency test; the 45 s means of transportation, musical instrument, and bird fluency test; and the 30 s vegetable, fruit, means of transportation, and musical instrument fluency test), and a score between 0.7 and 0.8 is considered acceptable (e.g., the 30 s bird fluency test).

### 3.4. Establishment of Test-Retest Reliability

This study randomly selected 29 participants from the healthy group (14 men and 15 women) and 30 participants from the DAT group (13 men and 17 women) for subsequent retests conducted two to four weeks after the initial test. The mean age of the retest participants from the healthy group (*M* = 72.07, *SD* = 5.33) was significantly lower than that of the retest participants from the DAT group (*M* = 77.83, *SD* = 6.61; *t* [57] = −3.68, *p* = 0.001). Additionally, no significant differences were observed in the mean years of schooling received by the retest participants between the healthy group (*M* = 9.97, *SD* = 3.46) and the DAT group (*M* = 9.80, *SD* = 5.05; *t* [51.41] = 0.15, *p* = 0.594).

The Pearson’s product–moment correlation coefficient analysis was conducted to examine the test–retest reliability of the semantic fluency test at test durations of 60, 45, and 30 s, and the results are presented in Table 7.

For a test duration of 60 s, the test–retest correlation analysis revealed significant correlations in the performance of the healthy group for all categories. The “birds” category exhibited a high correlation (*r* = 0.80, *p* < 0.001), whereas the “animals” (*r* = 0.56, *p* = 0.002), “fruits” (*r* = 0.55, *p* = 0.002), “vegetables” (*r* = 0.50, *p* = 0.005), “means of transportation” (*r* = 0.56, *p* = 0.002), and “musical instruments” (*r* = 0.51, *p* = 0.005) categories exhibited moderate correlations. In the DAT group, significant test–retest correlations were observed for all categories, with high correlations observed for the “animals” (*r* = 0.75, *p* < 0.001), “fruits” (*r* = 0.72, *p* < 0.001), “vegetables” (*r* = 0.89, *p* < 0.001), “birds” (*r* = 0.85, *p* < 0.001), “means of transportation” (*r* = 0.73, *p* < 0.001) and “musical instruments” (*r* = 0.74, *p* = 0.002) categories.

For a test duration of 45 s, significant test–retest correlation was observed in the performance of the healthy group for all categories. The “birds” category (*r* = 0.71, *p* < 0.001) exhibited a high correlation, whereas the “animals” (*r* = 0.59, *p* = 0.001), “fruits” (*r* = 0.51, *p* = 0.005), “vegetables” (*r* = 0.55, *p* = 0.002), “means of transportation” (*r* = 0.55, *p* = 0.002) and “musical instruments” (*r* = 0.49, *p* = 0.008) exhibited moderate test–retest correlations. In the DAT group, significant test–retest correlations were observed for all categories. The “animals” (*r* = 0.79, *p* < 0.001), “vegetables” (*r* = 0.87, *p* < 0.001), “birds” (*r* = 0.80, *p* < 0.001), and “musical instruments” (*r* = 0.76, *p* < 0.001) categories exhibited high correlations, whereas the “fruits” (*r* = 0.60, *p* < 0.001) and “means of transportation” (*r* = 0.65, *p* < 0.001) categories exhibited moderate test–retest correlations.

For a test duration of 30 s, no significant test–retest correlation was observed in the performance of the healthy group for the “fruits” (*r* = 0.29, *p* = 0.133) and “musical instruments” (*r* = 0.18, *p* = 0.361) categories, and moderate test–retest correlations were observed for the “animals” (*r* = 0.60, *p* = 0.001), “vegetables” (*r* = 0.55, *p* = 0.002), “birds” (*r* = 0.61, *p* < 0.001), and “means of transportation” (*r* = 0.59, *p* = 0.001) categories. In the DAT group, significant test–retest correlations were observed for all categories. The “vegetables” (*r* = 0.88, *p* < 0.001), “birds” (*r* = 0.84, *p* < 0.001), “animals” (*r* = 0.58, *p* = 0.001), “fruits” (*r* = 0.65, *p* < 0.001), “means of transportation” (*r* = 0.53, *p* = 0.003), and “musical instruments” (*r* = 0.64, *p* < 0.001) categories exhibited moderate correlations.

## 4. Discussion

This study aimed to investigate the differences in semantic fluency test performance between healthy older adults and patients with DAT in Taiwan, to examine the effects of demographic characteristics on semantic fluency scores, and to identify differences in semantic fluency scores obtained at different test durations. In addition, this study also established cutoff points for each category of semantic fluency tests with different test durations and test–retest reliability for semantic fluency tests.

The optimal cutoff points for the semantic fluency test with a test duration of 60 s were determined using the Youden index (*J*) [43] as follows: “animals (8/9)”, “fruits (6/7)”, “vegetables (5/6)”, “birds (3/4)”, “means of transportation (5/6)”, and “musical instruments (3/4)”. The tests had a sensitivity and specificity of 0.704–0.852 and 0.759–0.931, respectively. The cutoff points for semantic fluency tests with a test duration of 60 s obtained in this study were lower than those reported in other studies [25,46,47]. This may be attributed to the following reasons. First, the range of semantic categories differs across languages. Rosselli et al. [48] reported that the “vegetables” category in Spanish encompasses a broader range of plants, including fruits. For example, in Spanish, a lemon (limón) is considered a vegetable, whereas speakers of other languages generally classify a lemon as fruit. These variations in semantic categories across languages should be considered when comparing results from different studies. Second, different scoring standards used in each study can lead to variations in cutoff points. Third, interference from bilingualism can also affect performance on the tests. Rosselli et al. [48] compared the semantic fluency test performances of monolingual (i.e., participants speaking only English or Spanish) and bilingual participants (i.e., participants who spoke both English and Spanish). During the test, participants were only allowed to respond in one language. The study results revealed that the performance of bilingual participants was lower in the animal fluency test. This may be a result of possible interference between languages [48,49]. In the present study, the participants were instructed to respond in Mandarin Chinese. However, during the test process, participants often responded in other dialects and wasted time translating their responses to Mandarin Chinese, which may have led to the lower mean scores compared with other studies. However, despite the lower cutoff points, no significant differences were observed in the sensitivity and specificity of the semantic fluency tests between this study and other studies. The AUC values of the semantic fluency tests in this study were ≥0.80, indicating satisfactory discrimination ability despite the lower cutoff points.

Regarding the effects of different test durations on test scores, Mirandez et al. [37] reported significant differences in mean scores between patients without cognitive impairments and patients with mild cognitive impairments for the “animal” category within the first 15 s of testing and for the “fruits” and “means of transportation” categories within the first 30 s of testing. By contrast, the present study reported significant differences between the healthy group and the DAT group within the first 15 s of semantic fluency testing for all categories. The discrepancies between these findings may be attributed to the differences in patients’ cognitive impairment severity among the recruited participants. Mirandez et al. [37] included older adults with mild cognitive impairments, whereas the present study included older adults with mild DAT. Consequently, in the current study, significant differences in the performance between healthy older adults and patients with mild DAT were observed in a shorter test duration. In addition, test duration is not necessarily associated with test discriminatory power. The optimal cutoff point, sensitivity, specificity, and AUC of semantic fluency tests for different categories must be separately evaluated. Furthermore, researchers must consider whether adjustments to the demographic variables are necessary. When no adjustment was made to the demographic variables, the results of the analysis in the present study revealed that the 60 s animal fluency test exhibited the highest sum of sensitivity and specificity, which was followed by the 30 s animal fluency test, 45 s vegetable fluency test, 60 s fruit fluency test, 45 s animal fluency test, 45 s fruit fluency test, 60 s means of transportation fluency test, 60 s vegetable fluency test, 45 s musical instrument fluency test, and the 45 s means of transportation test. In summary, semantic fluency tests with the “birds” category exhibited the lowest discriminatory power.

Regarding test–retest reliability, studies on the 60 s semantic fluency test have reported moderate-to-high test–retest correlations in participants without cognitive impairments (*r* = 0.56–0.85). However, some participants exhibited higher retest scores than their initial test scores with some exhibiting significant improvements [19,20,24,33]. For a test duration of 60 s, the present study reported significant test–retest correlations in the performance of the healthy group for all categories (*r* = 0.50–0.80). Overall, at different test durations, the performance of the healthy group exhibited high or moderate test–retest correlation for most categories, with few categories exhibiting low or no correlation, including the 30 s fruit fluency test and 30 s musical instrument fluency test.

The performance of the DAT group in the 60 s semantic fluency test exhibited a high test–retest correlation for all categories. At most test durations, the test–retest correlation coefficient of the DAT group was higher than that of the healthy group. This finding is inconsistent with the literature, which indicates that the test–retest reliability of participants without cognitive impairments is higher than that of participants with DAT. Cooper et al. [24] conducted a study with a one-week interval between the initial test and retest and obtained a test–retest correlation of 0.50. They reported that 40% of participants in the DAT group had lower retest scores than initial test scores; however, the difference was not significant. The present study inferred that some of the participants in the DAT group had higher retest scores than initial test scores, leading to approximate mean scores between the two tests. However, the short-term test–retest reliability of the semantic fluency test requires further research with larger sample sizes. St-Hilaire et al. [22] employed a longer time interval (i.e., one year) between the initial test and retest of the semantic fluency test and reported a test–retest correlation of 0.493. They attributed the decline in overall performance to cognitive deterioration in participants. In addition, practice effects may also need to be discussed. In the present study, the retest scores of the healthy group in the 60 s semantic fluency tests were higher than the initial test scores. However, significant differences in test–retest scores were only observed in the “birds” and “means of transportation” categories, with a difference between the mean initial test scores and the mean retest scores of approximately one point. Similar results were obtained for other categories, with retest scores being approximately zero to two points higher than the initial test scores in most cases. However, no significant differences in test–retest scores were observed in the results of the 60 s semantic fluency tests in the DAT group. In some categories, the mean initial score was higher than, but not significantly different from, the mean retest score. For other test durations, the retest score was higher than the initial test score in some categories, whereas the initial test score was higher in other categories. In categories for which significant differences in the test–retest scores were observed, the differences between the mean initial test score and the mean retest score did not exceed one point. In summary, the test–retest reliability of the healthy group was lower than that of the DAT group, which was possibly due to the test interval of two to four weeks. Additionally, practice effects may have influenced the performance of healthy older adults, resulting in a higher retest performance in the “birds” and “means of transportation” categories. For older adults with DAT, no practice effect is observed within a two to four-week test–retest interval. Because the course of DAT is unlikely to rapidly deteriorate within two to four weeks, the performance score of older adults with DAT remains relatively stable, resulting in consistent retest performance.

The study has limitations that should be considered. First, the small sample size limited the ability of the study to recruit an equal number of participants for each age group and education level. Second, linguistic limitations may have affected the response performance of the participants. This current study specifically requested participants to respond in Mandarin Chinese during the test, which may have resulted in the lower cutoff point than that reported in the literature. Third, this current study only compared healthy adults without dementia with patients with DAT. Other types of dementia that may affect semantic fluency tests have not been studied, and the usefulness of the test in the screening of other types of dementia should also be examined in a future study.

## 5. Conclusions

This study explored the feasibility of using different semantic fluency test categories to distinguish between older adults without cognitive impairments and those with DAT. The categories used in the semantic fluency test were animals, fruits, vegetables, birds, means of transportation, and musical instruments. The scores of older adults without cognitive impairments and those with DAT for each category were analyzed. For older adults without cognitive impairments, gender, age, and education level were associated with scores in different categories of the semantic fluency test. By contrast, for older adults with DAT, among all demographic variables, the presence of DAT, characterized by deteriorating linguistic and cognitive abilities, had the greatest association with semantic fluency scores. Furthermore, this study proposed optimal cutoff points and calculated the AUC for various test durations and semantic categories in the semantic fluency test. These cutoff points and AUC values may serve as a reference that would help clinical personnel distinguish between older adults without cognitive impairments and those with DAT, thus enabling them to make accurate diagnosis.

## Figures and Tables

**Table 1:** Demographic characteristics of participants.

		Participant Group		*df*	*t*	*p*
		Healthy Group (*n* = 58)	DAT Group (*n* = 54)			
Gender	Men	28	23			
	Women	30	31			
Age	*M (SD)*	72.10 (5.79)	78.39 (6.68)	110	−5.33	<0.001
	Range	65–94	65–91			
Years of schooling	*M (SD)*	9.40 (3.34)	8.96 (5.01)	91.31	0.54	0.594
	Range	6–16	0–22			
MMSE	*M (SD)*	26.79 (1.85)	-	-	-	-
	Range	22–30	-			

Note: *M* = mean, *SD* = standard deviation. Dashes signify that information was not available.

**Table 2:** Participants’ scores in each category of the semantic fluency test for different test durations.

	Healthy Group (*n* = 58)	DAT Group (*n* = 54)	*df*	*t*	*p*
	Mean ± SD	Mean ± SD			
Test duration of 60 s					
Animals	12.48 ± 3.05	6.00 ± 3.27	110	10.86	<0.001
Fruits	10.50 ± 2.86	4.52 ± 2.53	110	11.69	<0.001
Vegetables	10.21 ± 3.86	4.19 ± 2.95	110	9.23	<0.001
Birds	5.16 ± 2.33	2.24 ± 2.05	110	7.01	<0.001
Means of transportation	7.03 ± 1.70	3.52 ± 1.92	110	10.26	<0.001
Musical instruments	6.60 ± 2.55	2.70 ± 2.00	110	9.04	<0.001
Test duration of 45 s					
Animals	11.12 ± 3.00	5.37 ± 2.84	110	10.40	<0.001
Fruits	9.40 ± 2.72	4.02 ± 1.95	103.34	12.09	<0.001
Vegetables	9.05 ± 2.90	3.81 ± 2.74	110	9.81	<0.001
Birds	4.64 ± 2.16	2.04 ± 1.87	110	6.79	<0.001
Means of transportation	6.36 ± 1.59	3.22 ± 1.81	110	9.79	<0.001
Musical instruments	5.93 ± 2.28	2.52 ± 1.85	110	8.66	<0.001
Test duration of 30 s					
Animals	9.50 ± 2.35	4.35 ± 2.44	110	11.37	<0.001
Fruits	7.52 ± 2.44	3.59 ± 1.99	110	9.30	<0.001
Vegetables	7.17 ± 2.52	2.94 ± 2.06	110	9.68	<0.001
Birds	3.93 ± 2.03	1.67 ± 1.65	110	6.45	<0.001
Means of transportation	5.71 ± 1.63	2.89 ± 1.62	110	9.16	<0.001
Musical instruments	5.02 ± 1.97	2.28 ± 1.98	110	7.35	<0.001

Note: SD = standard deviation.

**Table 3:** Results of multiple regression analysis of the effect of demographic variables on the semantic fluency scores for each category for a 60 s test duration (*n* = 112).

		*B*	*SE B*	*β*	*R^2^*	*Adj R^2^*	*F*
Animals	Whether the participant had DAT	−5.78 ***	0.65	−0.64 ***	0.57	0.55	34.78 ***
	Gender	−0.39	0.59	−0.04			
	Age	−0.10 *	0.05	−0.16 *			
	Years of schooling	0.21 **	0.08	0.19 **			
Fruits	Whether the participant had DAT	−5.40 ***	0.55	−0.67 ***	0.60	0.59	40.8 ***
	Gender	−1.32 *	0.50	−0.16 *			
	Age	−0.10 *	0.04	−0.17 *			
	Years of schooling	0.12	0.06	0.12			
Vegetables	Whether the participant had DAT	−5.61 ***	0.69	−0.62 ***	0.52	0.50	28.84 ***
	Gender	−2.53 *	0.63	−0.28 *			
	Age	−0.08	0.05	−0.13			
	Years of schooling	0.09	0.08	0.08			
Birds	Whether the participant had DAT	−2.31 ***	0.45	−0.44 ***	0.39	0.37	17.09 ***
	Gender	−0.06	0.41	−0.01			
	Age	−0.09 **	0.03	−0.23 **			
	Years of schooling	0.14 **	0.05	0.22 **			
Means of transportation	Whether the participant had DAT	−3.30 ***	0.39	−0.66 ***	0.51	0.49	27.49 ***
	Gender	0.05	0.35	0.01			
	Age	−0.03	0.03	−0.08			
	Years of schooling	0.07	0.04	0.12			
Musical instruments	Whether the participant had DAT	−3.27 ***	0.46	−0.55 ***	0.51	0.49	27.64 ***
	Gender	−0.07	0.42	−0.01			
	Age	−0.09 **	0.03	−0.20 **			
	Years of schooling	0.18 ***	0.05	0.26 ***			

Note: * *p* < 0.05. ** *p* < 0.01. *** *p* < 0.001.

**Table 4:** Results of multiple regression analysis of the effect of demographic variables on the semantic fluency scores for each category for a 45 s test duration (*n* = 112).

		*B*	*SE B*	*β*	*R^2^*	*Adj R^2^*	*F*
Animals	Whether the participant had DAT	−5.10 ***	0.61	−0.62 ***	0.53	0.52	30.68 ***
	Gender	−0.29	0.56	−0.04			
	Age	−0.10 *	0.04	−0.16 *			
	Years of schooling	0.15 *	0.07	0.15 *			
Fruits	Whether the participant had DAT	−4.82 ***	0.48	−0.67 ***	0.62	0.61	43.52 ***
	Gender	−1.26 **	0.44	−0.18 **			
	Age	−0.10 **	0.04	−0.19 **			
	Years of schooling	0.07	0.05	0.08			
Vegetables	Whether the participant had DAT	−4.84 ***	0.55	−0.63 ***	0.57	0.56	35.73 ***
	Gender	−2.40 ***	0.50	−0.31 ***			
	Age	−0.08 *	0.04	−0.15 *			
	Years of schooling	0.07	0.06	0.08			
Birds	Whether the participant had DAT	−2.05 ***	0.41	−0.43 ***	0.38	0.35	16.06 ***
	Gender	−0.13	0.38	−0.03			
	Age	−0.08 **	0.03	−0.23 **			
	Years of schooling	0.13 **	0.05	0.22 **			
Means of transportation	Whether the participant had DAT	−3.04 ***	0.36	−0.66 ***	0.48	0.46	24.60 ***
	Gender	−0.17	0.33	−0.04			
	Age	−0.01	0.03	−0.04			
	Years of schooling	0.07	0.04	0.12			
Musical instruments	Whether the participant had DAT	−2.82 ***	0.41	−0.53 ***	0.51	0.49	28.09 ***
	Gender	−0.11	0.37	−0.02			
	Age	−0.08 **	0.03	−0.21 **			
	Years of schooling	0.19 ***	0.05	0.29 ***			

Note: * *p* < 0.05. ** *p* < 0.01. *** *p* < 0.001.

**Table 5:** Results of multiple regression analysis of the effect of demographic variables on the semantic fluency scores for each category for a 30 s test duration (*n* = 112).

		*B*	*SE B*	*β*	*R^2^*	*Adj R^2^*	*F*
Animals	Whether the participant had DAT	−4.59 ***	0.50	−0.66 ***	0.57	0.56	36.00 ***
	Gender	0.07	0.46	0.01			
	Age	−0.08 *	0.04	−0.16 *			
	Years of schooling	0.11	0.06	0.13			
Fruits	Whether the participant had DAT	−3.36 ***	0.44	−0.57 ***	0.55	0.53	32.07 ***
	Gender	−1.58 ***	0.40	−0.27 ***			
	Age	−0.10 **	0.03	−0.23 **			
	Years of schooling	0.08	0.05	0.11			
Vegetables	Whether the participant had DAT	−3.87 ***	0.46	−0.62 ***	0.55	0.53	32.73 ***
	Gender	−1.75 ***	0.42	−0.28 ***			
	Age	−0.07 *	0.03	−0.16 *			
	Years of schooling	0.05	0.05	0.07			
Birds	Whether the participant had DAT	−1.80 ***	0.38	−0.42 ***	0.35	0.32	14.15 ***
	Gender	−0.09	0.35	−0.02			
	Age	−0.07 *	0.03	−0.22 *			
	Years of schooling	0.11 **	0.04	0.22 **			
Means of transportation	Whether the participant had DAT	−2.70 ***	0.34	−0.63 ***	0.46	0.44	22.47 ***
	Gender	−0.05	0.32	−0.01			
	Age	−0.01	0.03	−0.05			
	Years of schooling	0.08 *	0.04	0.16 *			
Musical instruments	Whether the participant had DAT	−2.29 ***	0.40	−0.48 ***	0.42	0.40	19.48 ***
	Gender	−0.52	0.36	−0.11			
	Age	−0.07 *	0.03	−0.19 *			
	Years of schooling	0.16 ***	0.04	0.28 ***			

Note: * *p* < 0.05. ** *p* < 0.01. *** *p* < 0.001.

**Table 6:** Optimal cutoff point and AUC of each semantic category for different test durations.

	Optimal Cutoff Point	Sensitivity	Specificity	AUC (*SD*)	AUC 95% CI	*p*
Test duration of 60 s						
Animals	8/9	0.815	0.931	0.93 (0.025)	0.88–0.97	<0.001
Fruits	6/7	0.796	0.914	0.94 (0.022)	0.89–0.98	<0.001
Vegetables	5/6	0.741	0.931	0.90 (0.030)	0.84–0.96	<0.001
Birds	3/4	0.741	0.793	0.83 (0.039)	0.76–0.91	<0.001
Means of transportation	5/6	0.852	0.828	0.91 (0.027)	0.86–0.96	<0.001
Musical instruments	3/4	0.741	0.914	0.89 (0.032)	0.83–0.95	<0.001
Test duration of 45 s						
Animals	8/9	0.889	0.810	0.92 (0.025)	0.87–0.97	<0.001
Fruits	6/7	0.852	0.845	0.94 (0.019)	0.90–0.98	<0.001
Vegetables	5/6	0.815	0.897	0.91 (0.029)	0.85–0.96	<0.001
Birds	2/3	0.648	0.845	0.82 (0.040)	0.74–0.90	<0.001
Means of transportation	5/6	0.870	0.776	0.90 (0.029)	0.84–0.96	<0.001
Musical instruments	3/4	0.741	0.879	0.88 (0.032)	0.82–0.95	<0.001
Test duration of 30 s						
Animals	7/8	0.889	0.828	0.94 (0.021)	0.89–0.98	<0.001
Fruits	6/7	0.907	0.690	0.89 (0.030)	0.83–0.95	<0.001
Vegetables	4/5	0.722	0.897	0.90 (0.028)	0.85–0.95	<0.001
Birds	2/3	0.722	0.776	0.80 (0.042)	0.72–0.89	<0.001
Means of transportation	4/5	0.815	0.810	0.88 (0.031)	0.82–0.94	<0.001
Musical instruments	2/3	0.630	0.931	0.84 (0.037)	0.77–0.92	<0.001

Note: *SD* = standard deviation, CI = confidence interval.

**Table 7:** Test–retest reliability of the semantic fluency test in the two groups of participants for different test durations.

	Healthy Group (*n* = 29)	DAT Group (*n* = 30)
Test duration of 60 s		
Animals	0.56 **	0.75 ***
Fruits	0.55 **	0.72 ***
Vegetables	0.50 **	0.89 ***
Birds	0.80 ***	0.85 ***
Means of transportation	0.56 **	0.73 ***
Musical instruments	0.51 **	0.74 ***
Test duration of 45 s		
Animals	0.59 **	0.79 ***
Fruits	0.51 **	0.60 ***
Vegetables	0.55 **	0.87 ***
Birds	0.71 ***	0.80 ***
Means of transportation	0.55 **	0.65 ***
Musical instruments	0.49 **	0.76 ***
Test duration of 30 s		
Animals	0.60 **	0.58 **
Fruits	0.29	0.65 ***
Vegetables	0.55 **	0.88 ***
Birds	0.61 ***	0.84 ***
Means of transportation	0.59 **	0.53 **
Musical instruments	0.18	0.64 ***

Note: ** *p* < 0.01. *** *p* < 0.001.

## Data Availability

The data generated during and/or analyzed during the current study are available from the corresponding author on reasonable request.
